# Persistent inequities in maternal mortality in Latin America and the Caribbean, 1990–2019

**DOI:** 10.1186/s12939-024-02100-y

**Published:** 2024-05-10

**Authors:** Rocío Sáenz, Gustavo Nigenda, Ingrid Gómez-Duarte, Karol Rojas, Arachu Castro, Edson Serván-Mori

**Affiliations:** 1 Center for Research in Nursing and Health Care (CICES), University of Costa Rica, San Pedro Montes de Oca, San Jose, Costa Rica; 2 Faculty of Nursing and Obstetrics, National Autonomous University of Mexico, Mexico City, Mexico; 3 Department of International Health and Sustainable Development, School of Public Health and Tropical Medicine, Tulane University, New Orleans, State of Louisiana USA; 4 Center for Health Systems Research, National Institute of Public Health of Mexico, Universidad Av. 655, Cuernavaca, Morelos Mexico

**Keywords:** Equity, Maternal mortality, Government health expenditure, Human resources for health, Maternal health coverage

## Abstract

**Background:**

Despite the resources and personnel mobilized in Latin America and the Caribbean to reduce the maternal mortality ratio (MMR, maternal deaths per 100 000 live births) in women aged 10–54 years by 75% between 2000 and 2015, the region failed to meet the Millenium Development Goals (MDGs) due to persistent barriers to access quality reproductive, maternal, and neonatal health services.

**Methods:**

Using 1990–2019 data from the Global Burden of Disease project, we carried out a two-stepwise analysis to (a) identify the differences in the MMR temporal patterns and (b) assess its relationship with selected indicators: government health expenditure (GHE), the GHE as percentage of gross domestic product (GDP), the availability of human resources for health (HRH), the coverage of effective interventions to reduce maternal mortality, and the level of economic development of each country.

**Findings:**

In the descriptive analysis, we observed a heterogeneous overall reduction of MMR in the region between 1990 and 2019 and heterogeneous overall increases in the GHE, GHE/GDP, and HRH availability. The correlation analysis showed a close, negative, and dependent association of the economic development level between the MMR and GHE per capita, the percentage of GHE to GDP, the availability of HRH, and the coverage of SBA. We observed the lowest MMRs when GHE as a percentage of GDP was close to 3% or about US$400 GHE per capita, HRH availability of 6 doctors, nurses, and midwives per 1,000 inhabitants, and skilled birth attendance levels above 90%.

**Conclusions:**

Within the framework of the Sustainable Development Goals (SDGs) agenda, health policies aimed at the effective reduction of maternal mortality should consider allocating more resources as a necessary but not sufficient condition to achieve the goals and should prioritize the implementation of new forms of care with a gender and rights approach, as well as strengthening actions focused on vulnerable groups.

## Background

Sustained progress toward health equity is essential for the adequate performance of health systems [1, 2] to achieve the aspirations of universal health coverage (UHC), particularly in low- and middle-income countries [3, 4]. Despite the progress observed in these countries since the Millennium Development Goals (MDGs) went into effect in 2000, a blueprint agreed to by all the world’s countries and leading development institutions, —by which all world countries agreed to improve maternal health by reducing the maternal mortality ratio (MMR) by 75% and achieve universal access to reproductive health by 2015, among other commitments—, health inequities persist among the most socially disadvantaged populations [5, 6]. This occurs mainly in countries that have failed to achieve universality of health care, including some in the Latin American and Caribbean region (LAC) [7–9].

Maternal mortality reflects the inability of health systems to ensure effective access to essential health services [10–13] and is one of the most sensitive indicators of the persistence of health inequities [14–17] and the social inequality that societies experience [18]. The social determinants of health are the social conditions into which people are born, grow, live, work, and age [17]; the structural determinants, in turn, shape the distribution of social determinants; the multiple pathways between structural and social determinants can lead to higher maternal and infant mortality rates and socially defined inequities [12]. Maternal health policies and programs implemented in LAC over the past three decades have brought with them modest and heterogeneous progress in reducing maternal mortality [11, 19, 20] that is at risk of being lost due to the effects of the COVID-19 pandemic [21].

Public policymakers have emphasized the need to review and guide the actions undertaken by the countries in the region to analyse the design, implementation, and viability of health policies; and increase their capacity to reduce the structural mechanisms of exclusion from effective access to health services (e.g., structural discrimination) [22]. Similarly, they have emphasized the reduction of the asymmetries of economic and social power that would allow the modification of their structural causes to effectively close the gaps in the economic, social, cultural, and political inequality of women and, at the same time, favour the protection of vulnerable populations [23]. A key element in this change is strengthening the timely, efficient, and equitable response capacity of health systems and services, including allocating required human, financial, and technological resources [16, 19].

Guiding the actions to be undertaken in the immediate future and the medium and long term requires updated and comprehensive information on LAC countries’ performance in sustainably and effectively reducing maternal mortality. However, few regional studies primarily focused on analysing observed trends in maternal mortality document this situation [21, 24–27]. This study aimed: (1) To describe the differences in the MMR temporal patterns (1990–2019) in the LAC region, (2) to assess the relationship between MMR and selected indicators: government health expenditure (GHE), GHE as a percentage of gross domestic product (GDP), the availability of human resources for health (HRH), the coverage of effective interventions to reduce maternal mortality, and the level of economic development of each country.

## Methods

### Design and data

We conducted a multi-country longitudinal study for LAC during the period 1990–2019. We analysed secondary data from the Global Burden of Disease (GBD) study [28], publicly available at http://ghdx.healthdata.orgsite. Following the GATHER guidelines [29], this study generates historical information on the burden of disease at the country level. These data’s methodological details and analytical scope can be consulted in other publications [28, 30]. Of the 33 countries in the region, we excluded Saint Kitts and Nevis due to a lack of data in all the indicators analysed.

### Variables

We evaluated the MMR (maternal deaths per 100 000 live births) in women aged 10–54 years in terms of government health expenditure per capita (in thousands of US$ of purchasing power parity 2020); GHE as a percentage of gross domestic product (GDP) (GHE/GDP); availability of HRH (number of physicians, nurses and midwives, and pharmacists per 1,000 population); percentage coverage of births attended by skilled health personnel (SBA); and a socio-demographic index (SDI) (range 0–1) as an economic development proxy variable of each country [31]. The SDI is calculated as the geometric mean of the total fertility rate before the age of 25, the average schooling for those over 15 years of age, and the per capita income. Higher SDI values reflect greater development relevant to health [31]. We classified countries into five levels of development in 1990 (baseline) using the Dalenius-Hodges method [32].

### Statistical analysis

We performed data processing and analysis using the Stata statistical package version 17MP. We describe the differences in the temporal patterns (1990–2019) of the MMR in the LAC region, presenting the smoothed temporal evolution of the indicators of interest (considering a bandwidth = 0.6) adjusted for annual fixed effects [33]; and the levels and percentage change (relative change between 1990 and 2015 and 1990–2019, and the average annual growth rate 1990–2019) of MMR in analyzed countries ranked according to baseline (1990) SDI level. We emphasized our description in two relevant moments of the period analyzed: 2000 —the beginning of the MDGs era—, and 2015 —the end of the MDGs and the beginning of the SDG era—.

To evaluate the relationship between the MMR and the indicators mentioned previously, we first graphed the results estimated by nonlinear regression models with polynomial-fractional function [34] (with 95% CI) for the association between MMR, GHE, HRH availability, and SBA coverage. We adjusted these models by country and annual fixed effects and evaluated their goodness of fit using the respective variation coefficients (*R*^*2*^). Each country was represented by a circle weighted by the size of the female population aged 10–54 years concentrated in each country (as a proxy for potential demand for maternal health services). Second, we analysed the evolution between 1990 and 2019 of absolute inequality in the MMR by SDI level (also emphasizing the years 2000 and 2015 as they relate to the MDGs and SDGs). Third, we conducted a performance analysis of national health systems in terms of GHE/GDP (input) and MMR (result). To this end, we defined performance quadrants, placing the standardized average annual exchange rate of input (GHE/GDP) on the abscissa axis and the standardized average annual exchange rate of the result of interest (MMR) on the ordinate axis. Thus, each quadrant combines investment and health achievements, suggesting areas in which a country should focus. We categorized countries according to their SDI level.

## Results

**Fig. 1:**
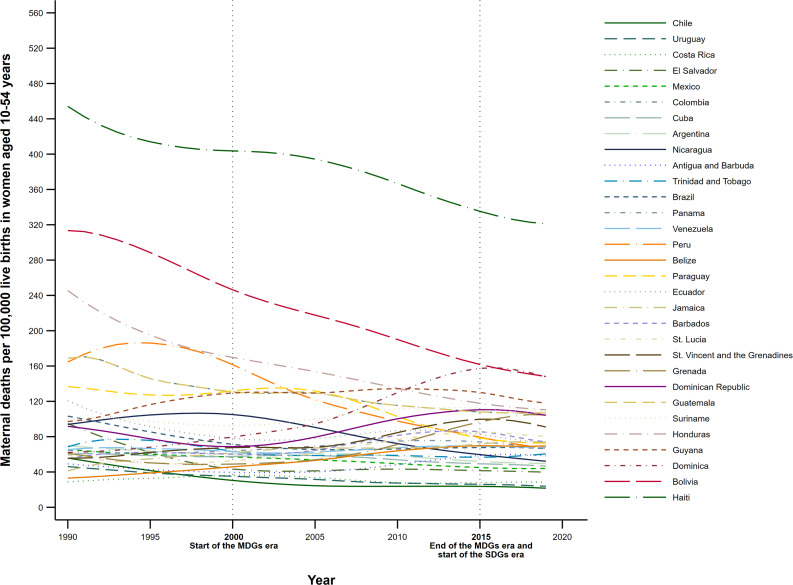
Temporal trends in the maternal mortality ratio (MMR) in Latin American and Caribbean countries, 1990–2019. (Note: Elaborated by the authors using data from the Global Burden of Disease study [72])

We observed a sustained overall decline in MMR, with stagnation patterns during the last years of the MDG period (2010–2015) extending to the first five years of the SDG period (Fig. 1). However, Haiti stood out as an isolated case, registering triple-digit levels, 20 times higher than the lowest observed in the region, followed by Bolivia, Dominica, Guyana, Suriname, and Honduras. In contrast, Chile, Uruguay, and Costa Rica were the three countries with the lowest MMR (< 30). Some English-speaking Caribbean countries recorded significant MMR increases, most notably Dominica, with an increase of 134.0% from 1990 to 2019. In this period, Chile, Peru, and El Salvador registered the largest reductions (63.3%, 59.7%, and 58.2%, respectively), although in smaller quantity concerning the 2015 goals, and which were equivalent for 2019 to MMRs of 21.8, 69.1, and 40.2 respectively. These three countries also recorded the highest rate of MMR reduction (3.3%, 3.0%, and 2.9% per year), followed by Honduras (2.6%) and Bolivia (2.3%) (Table 1).

**Table 1:** Levels and change of maternal mortality rate in Latin American and Caribbean countries, 1990–2019

LAC countries according to baseline (1990) SDI level	Maternal deaths per 100,000 live births in women aged 10–54 years				Relative change (%)		Average annual growth rate (%)
	1990	2000	2015	2019	1990–2015	1990–2019	
***Lowest***							
El Salvador	96.4	38.3	41.9	40.2	-56.6	-58.2	-2.9
Guatemala	160.4	134.9	93.1	106.4	-42.0	-33.7	-1.4
Haiti	460.5	407.4	330.4	326.2	-28.2	-29.2	-1.1
Honduras	248.7	170.0	116.2	111.5	-53.3	-55.2	-2.6
Nicaragua	93.0	106.1	59.8	53.8	-35.7	-42.2	-1.8
***Low***							
Belize	33.2	42.8	73.6	73.0	121.5	119.8	2.7
Bolivia	302.2	236.8	159.6	148.6	-47.2	-50.8	-2.3
Dominican Republic	92.0	60.5	117.0	103.2	27.2	12.2	0.4
Guyana	111.4	124.5	128.6	119.2	15.5	7.0	0.2
***Middle***							
Brazil	106.1	68.4	69.1	66.3	-34.8	-37.5	-1.6
Colombia	67.6	75.7	76.7	49.0	13.5	-27.5	-1.1
Ecuador	124.8	98.1	82.2	74.3	-34.2	-40.5	-1.7
Grenada	62.4	49.1	99.7	99.6	59.9	59.7	1.6
Paraguay	135.4	129.9	73.5	73.2	-45.7	-45.9	-2.0
Peru	171.4	162.4	72.6	69.1	-57.7	-59.7	-3.0
Saint Lucia	60.0	48.9	85.0	81.4	41.6	35.5	1.0
Saint Vincent and the Grenadines	53.9	69.7	116.5	89.5	116.4	66.2	1.7
Suriname	98.3	82.0	107.7	111.2	9.5	13.0	0.4
***High***							
Costa Rica	25.2	39.1	28.3	28.5	12.6	13.4	0.4
Jamaica	42.3	62.8	76.1	73.6	80.0	74.2	1.9
Mexico	67.1	56.7	43.6	44.5	-35.0	-33.7	-1.4
Panama	65.9	59.7	73.3	66.0	11.3	0.2	0.01
Venezuela	67.1	61.4	68.1	68.1	1.5	1.5	0.05
***Highest***							
Antigua and Barbuda	49.9	37.5	67.9	58.0	36.2	16.3	0.5
Argentina	70.3	55.5	52.5	49.2	-25.2	-29.9	-1.2
Barbados	57.3	52.7	84.0	75.0	46.6	30.7	0.9
Chile	59.3	29.1	25.4	21.8	-57.1	-63.3	-3.3
Cuba	59.1	54.3	53.4	46.2	-9.8	-21.9	-0.8
Dominica	63.3	80.5	179.4	148.7	183.3	134.8	2.9
Trinidad and Tobago	70.5	68.5	54.9	62.2	-22.1	-11.8	-0.4
Uruguay	36.6	31.6	26.9	23.9	-26.4	-34.6	-1.4

Note: Elaborated by the authors using data from the Global Burden of Disease study [72]

**Fig. 2:**
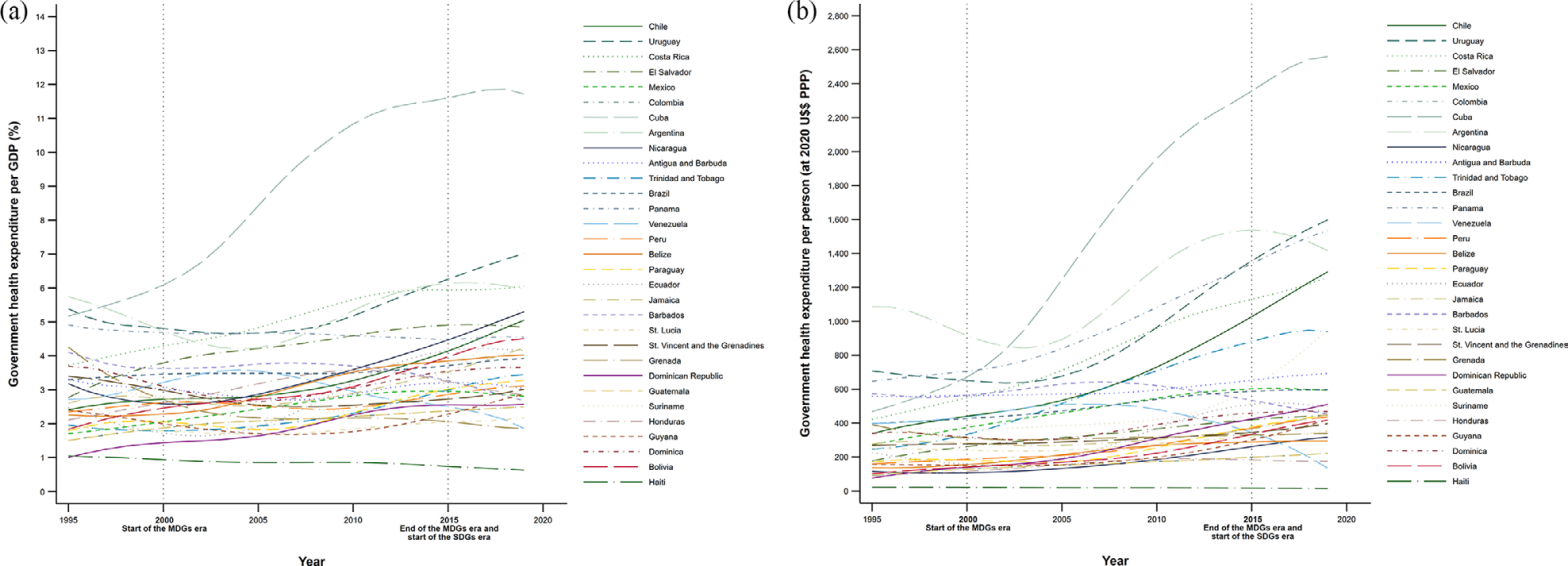
Temporal trends in government health expenditure (GHE) in Latin American and Caribbean countries, 1995–2019. Panel **a**. GHE per GDP. Panel **b**. GHE per person. (Note: Elaborated by the authors using data from the Global Burden of Disease study [72])

National GHEs grew steadily according to their development level (Fig. 2). The countries with the highest growth from 1995 to 2019 in the GHE as a percentage of GDP were the Dominican Republic (160.0%, from 1.0 to 2.6%), Bolivia (144.0%, from 1.8 to 4.4%), Cuba (125.0%, from 5.2 to 11.7%), Chile (108.0%, from 2.4 to 5.0%), and Suriname (90.0%, from 3.0 to 5.7%). In contrast, the countries that reduced this percentage in the same period were Venezuela (58.6%, from 2.9 to 1.2%), Grenada (53.7%, from 4.1 to 1.9%), Haiti (45.5%, from 1.1 to 0.6%), Barbados (32.5%, from 4.0 to 2.7%), and Dominica (18.9%, from 3.7 to 3.0%) (Fig. 2, Panel a). From 1995 to 2019, the countries that increased their GHE per capita the most were the Dominican Republic (567.5%, from US$77 to US$514), Cuba (450.9%, from US$464 to US$2,556), Bolivia (314.3%, from US$98 to US$406), Chile (289.0%, from US$328 to US$1,276), and Trinidad and Tobago (263.7%, from US$251 to US$913); while the countries that reduced the level of their GHE per capita were Venezuela (81.6%, from US$418 to US$77), Haiti (43.5% from US$23 to US$13), and Barbados (18.0%, from US$551 to US$452). In 2019, Cuba stood out for its higher GHE per capita (US$2,556), followed by Uruguay (US$1,581), Panama (US$1,482), Argentina (US$1,384), and Costa Rica (US$1,297) (Fig. 2, Panel b).

**Fig. 3:**
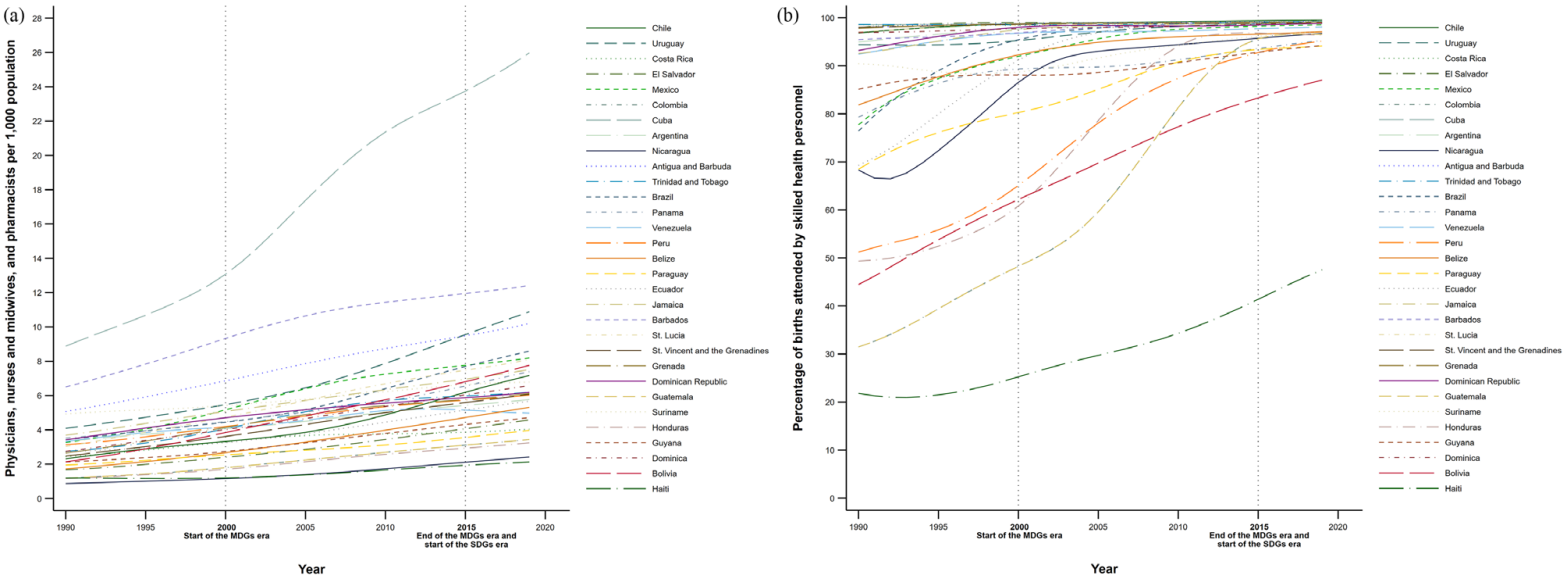
Temporal trends in the availability of human resources for health (HRH) and in the coverage of skilled birth attendance (SBA) in Latin American and Caribbean countries, 1990–2019. Panel **a**. Availability of HRH. Panel **b**. Coverage of SBA. (Note: Elaborated by the authors using data from the Global Burden of Disease study [72])

The increasing trend in GHE and its heterogeneous patterns corresponded with the overall growth in HRH availability and SBA coverage (Fig. 3). From 1990 to 2019, the countries that increased their HRH availability by the highest percentage were Bolivia (260.5%), Chile (214.7%), Belize (209.4%), Cuba (198.3%), and Guatemala (186.2%); while the countries with the lowest growth in this indicator were Suriname (40.3%), Venezuela (52.7%), Costa Rica (65.7%), Argentina (73.0%), and Haiti (79.8%). In 2019, Cuba was the country with the highest availability of HRH in the region, with 26.2 doctors, nurses, and midwives per 1,000 inhabitants, followed by Barbados (12.5), Uruguay (10.9), and Antigua and Barbuda (10.2). On the other hand, the countries with the lowest availability of HRH in 2019 were Haiti (2.1), Nicaragua (2.4), Honduras (3.3), Guatemala (3.5), and Paraguay (4.0) (Fig. 3, Panel a). We also observed a clear regional convergence towards universality in SBA coverage, except for Haiti (which registered a coverage level in 2019 of 47.3%), with notable growths such as those registered in Guatemala (from 32.1% in 1990 to 96.8% in 2019), Honduras (49.2–96.7%), Bolivia (45.1–87.0%), and Peru (51.2–85.2%) (Fig. 3, Panel b).

**Fig. 4:**
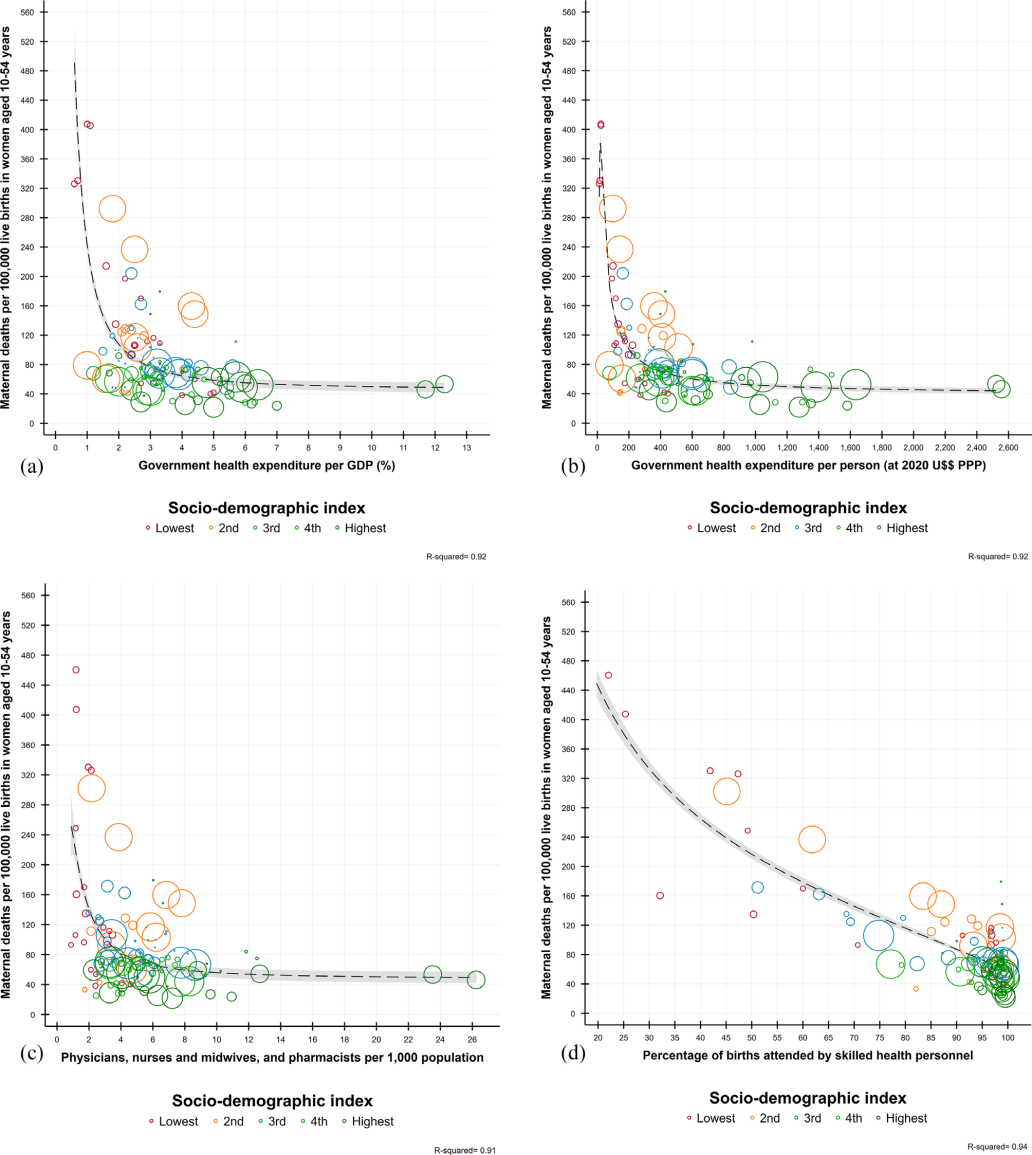
Association between the maternal mortality ratio (MMR), government health expenditures (GHE), availability of human resources for health (HRH) and the coverage of skilled birth attendance (SBA) in Latin American and Caribbean countries, 1990–2019. Panel **a**. MMR and GHE per GDP. Panel **b**. MMR and GHE per person. Panel **c**. MMR and availability of HRH. Panel **d**. MMR and SBA. (Note: Elaborated by the authors using data from the Global Burden of Disease study [72])

**Fig. 5:**
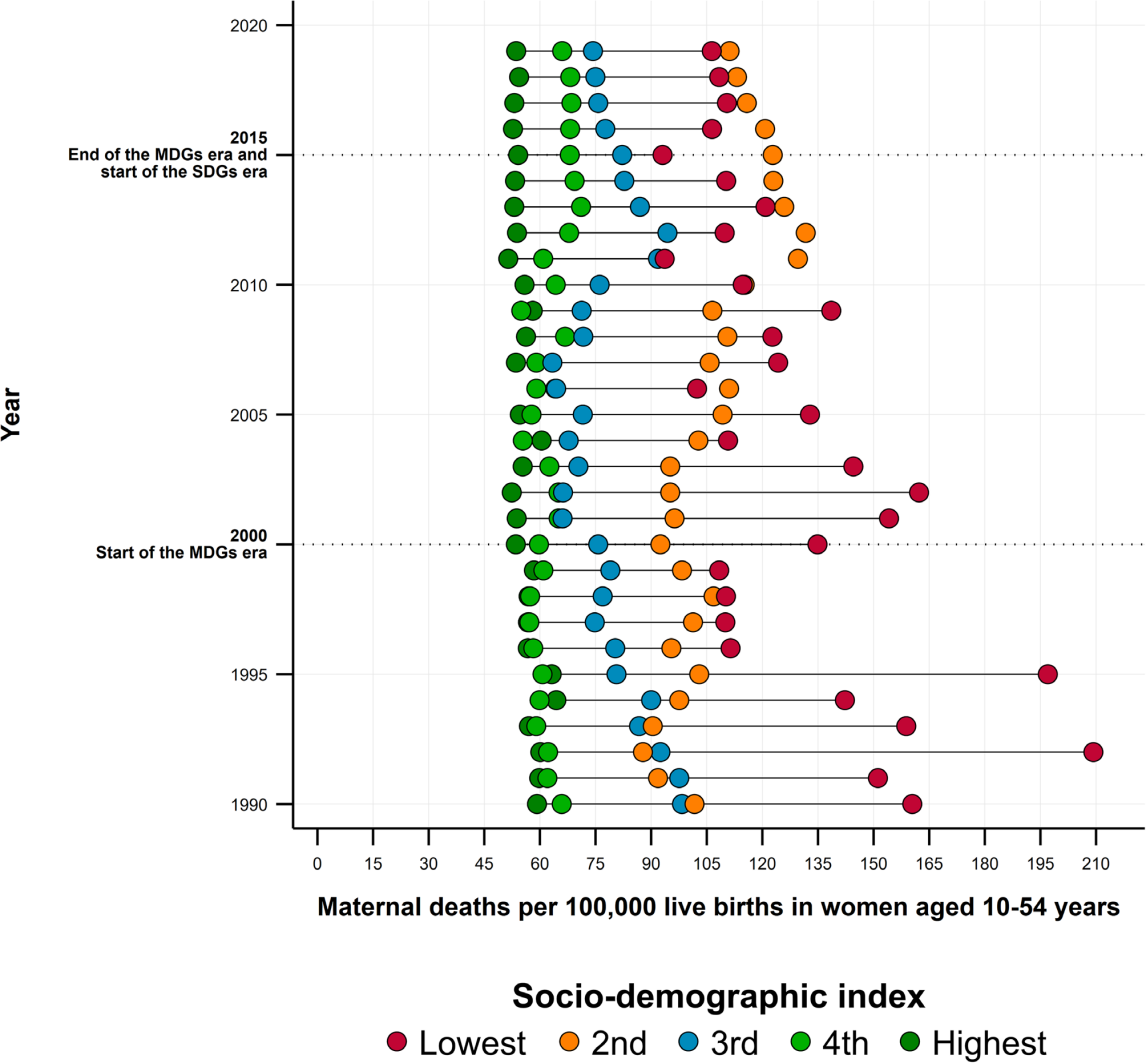
Equity trends in maternal mortality ratio (MMR) according to the socio-demographic index (SDI) in Latin America and Caribbean countries, 1990–2019. (Note: Elaborated by the authors using data from the Global Burden of Disease study [72])

The correlation analyses showed a close, negative, and dependent association of the economic development level between the MMR and GHE per capita, the percentage of GHE to GDP, the availability of HRH, and the coverage of SBA (Fig. 4, Panels a-d). We observed the lowest MMRs when GHE as percentage of GDP was close to 3% or about US$400 GHE per capita, HRH availability of 6 doctors, nurses, and midwives per 1,000 inhabitants, and SBA coverage levels above 90%. We also observed a general trend toward reducing inequities in the MMR between the region’s countries, especially during the MDG era and between those located at medium, high, and very high levels of economic development (Fig. 5). This trend was broken toward the end of the MDGs and the beginning of the SDGs, delineating two large groups associated with their development level: (a) countries that in 1990 were at high or very high development levels, with similar levels of MMR, and (b) countries that at baseline were at medium, low or very low development levels, characterized by their convergence towards higher MMRs.

**Fig. 6:**
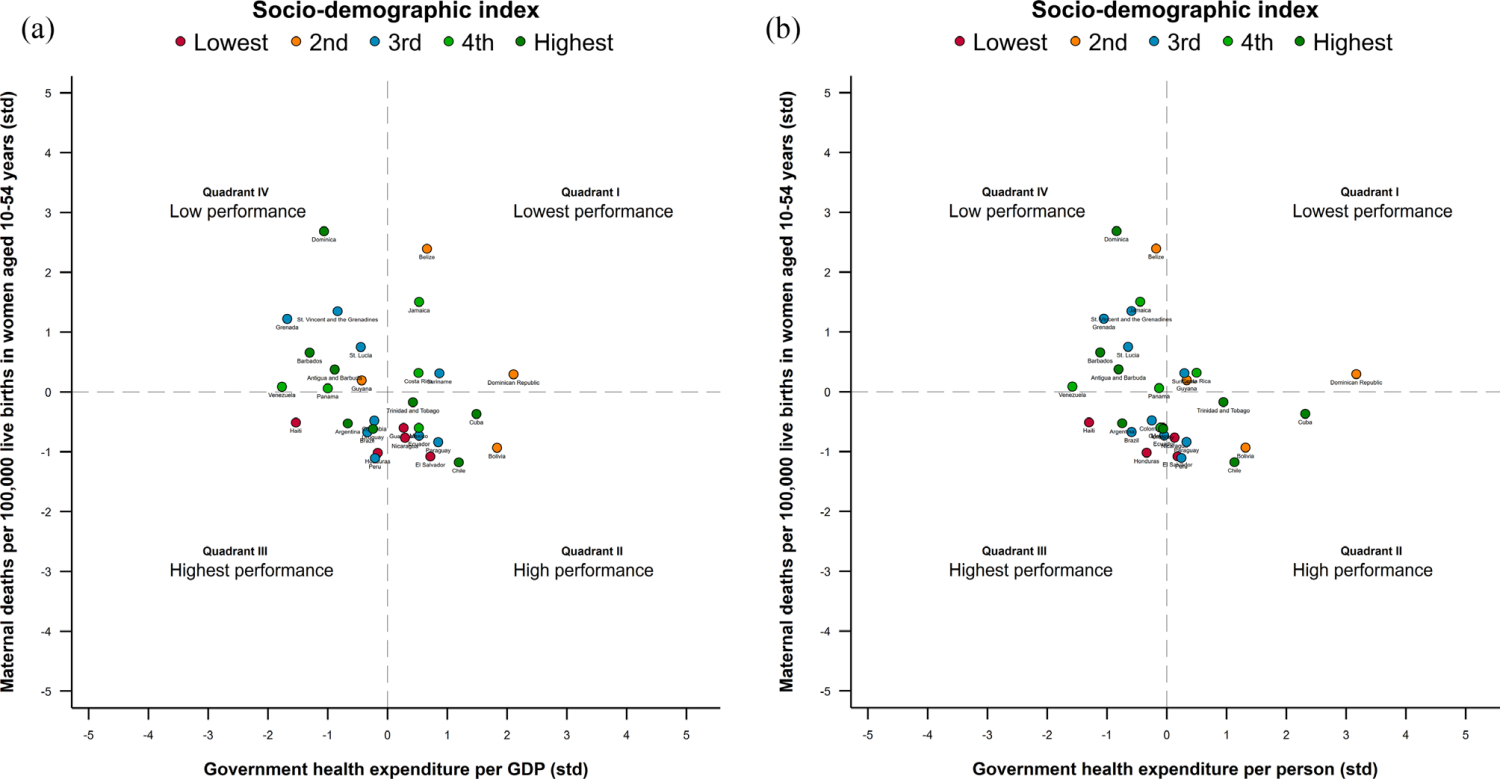
Performance analysis results: Annual average exchange rate of maternal mortality ratio (MMR, standarized) and government health expenditures (GHE, standarized) in Latin American and Caribbean countries, 1990–2019. Panel **a**. MMR and GHE per GDP. Panel **b**. MMR and GHE per person. (Note: Elaborated by the authors using data from the Global Burden of Disease study [72])

Figure 6 (Panel a and b) shows the results of the quadrant performance analysis. 22.6% of LAC countries were in the lowest performance quadrant (higher increase of MMR and GHE), including Belize, Costa Rica, Dominican Republic, Guyana, Panama, Suriname, and Trinidad and Tobago; 25.8% of countries were in the low performance quadrant (higher increase in MMR and lower increase in GHE); 35.5% of countries were in the high performance quadrant (greater reduction in the MMR and increase of the GHE); and 16.1% of countries were located in the highest performance quadrant (greater reduction in the MMR and lower increase of the GHE), highlighting Argentina, Brazil, Honduras, Colombia, and Haiti.

## Discussion

The main results can be summarized as follows. Over the study period in the LAC region, we observed: (1) a general decline of MMR, (2) a notable increase in GHE and its percentage of GDP, (3) a remarkable increase in HRH availability, (4) a remarkable increase in the coverage of skilled birth attendance.

The decline in the MMR observed in LAC enabled a small group of countries to achieve MDG 5, consisting in reducing the MMR by 75% between 1990 and 2015. However, while we observed a trend towards a reduction of the MMR during the MDG era, differences persist across social groups, being more noticeable towards the beginning of the SDG era.

The reduction of MMR in the MDG period could be attributed to various interventions undertaken from 2000 to 2015, both systemic (such as strengthening the accessibility to contraceptive methods, preventing unwanted pregnancies, increasing universal access to maternal health services, strengthening information and monitoring systems, increasing financial resources, or legalizing the termination of pregnancies) and those focused on improving clinical practice in maternal health facilities (such as the training of health personnel for prenatal, childbirth and postpartum care, the correct use of protocols, the redesign of reference and counter-reference systems, the promotion of quality of care, and the prevention of infections, mainly) [35], in several countries [36–40]. Haiti also reduced its MMR in 2019 but is still more than 170 points behind the second country with the highest MMR (Dominica with an MMR = 148.7). This situation has been attributed to Haiti's historical process of a chronic political crisis, its difficulty in implementing sustainable social policies, and the scarcity of human resources for maternal health care [41, 42].

The increase in GHE and its percentage of GDP observed in the region, framed in a period of low economic growth (around 2% per year for the region) [43], can be related to the effect of the 1990s reforms that involved the mobilization of public resources to expand coverage and the provision of resources to the system [44, 45] as well as the focalization of a significant amount of resources to comply with the MDGs related to the reduction of maternal mortality (MDG 5) [46]. The increase in HRH availability in the region was also remarkable, mainly in Bolivia, Chile, and Belize, with increases of 260.5%, 214.7%, and 209.4% respectively. English-speaking Caribbean countries, which have traditionally had a large availability of HRH, particularly nurses, managed to increase their availability further, approaching the WHO recommendation of 23 health workers per 10,000 inhabitants.

In addition to the increase in HRH density, countries in the region increased the coverage of skilled birth attendance, reaching more than 90% since 2000, regardless of the volume of potential demand for maternal health services. Guatemala and Honduras showed extraordinary growth, particularly Guatemala, to attend more than 95% of births with qualified personnel, followed by Bolivia and Peru, with coverage levels greater than 85%. This implies that most of the countries in the region have made an enormous effort to ensure that childbirth care is provided by qualified personnel, regardless of the existence of different strategies to achieve this goal. However, without prejudice to the effort made, the distribution of these human resources by geographical region within each country and socio-demographic groups still show significant inequalities [47].

We found that both GHE and HRH availability is a necessary but not a sufficient condition for achieving health goals and equity in resource allocation. Both indicators had an inverse and asymptotic relationship with the MMR, registering (for the first case) the minimum MMR of around 50 and US$3,000. For the second, a minimum MMR about 75 with a density of HRH around 5, evidencing the limits of investment and the possibility that other variables in the provision, organization, management, or HRH endowment may be involved in a greater health gain. This suggests the need to strengthen social protection systems and implement intersectoral and inclusive social policies that transcend the health system and link other sectors to address social and economic structural causes, focusing on the most vulnerable and underserved populations.

Equity as a guide in distributing government resources for effective health care constitutes, in essence, a mechanism for the redistribution of these resources in society. It is important to note that the complementarity of social and health investment makes it possible to confront the false dichotomy between health efficiency and social equity. While some authors raise the possibility of this conflict [48], others consider it a fallacy [49]. We argue that equity should guide the development of effective public policies.

However, the results of these efforts to expand resources were heterogeneous. The quadrant performance analysis showed that insufficient public investment in about 40% of countries, especially in the English-speaking Caribbean, resulted in smaller reductions in the MMR. In contrast, countries such as Cuba, Chile, and Peru obtained the most significant reductions from budget increases above the regional average, noting that the increase in GHE faced challenges that could not be met by translating the availability of these resources into adequate care models to address a complex phenomenon with structural determinants as profound as maternal mortality. In financial terms, a small portion of countries saw their public spending grow exponentially, while in more than half, the growth was moderate. This situation raises the need to innovate in the design and implementation of policies, programs, and strategies that consider the incorporation of approaches focused on users and their needs, with a focus on human rights, gender, and interculturality to achieve quality care for all women, regardless of their ethnic affiliation or socioeconomic position. Likewise, it is essential to implement mechanisms to monitor their progress and to make adjustments to achieve the proposed objectives [45].

The main teaching of our results is that we need to move beyond the sole allocation of resources to implement innovative strategies and policies. First, health equity policies must include in their design a holistic view of the reality they face [50]; that is, they must incorporate criteria of relevance of services and information on the social, demographic, and historical profile [51] of the target populations [52]. Second, these policies should also seek a balance between accessibility and quality [53] and a correct combination of incentives and regulation that improves the quality of care while implementing mechanisms for effective financing, stewardship, empowerment, and exercise of sexual and reproductive rights [54, 55], ensuring timely and respectful access to maternal health services, particularly for population sectors with the greatest lags in health outcomes. Likewise, investment in education for women and the possibility of entering the labor market must be part of a package of policies targeting different inequality determinants in the region’s populations.

There is no point in strengthening the availability and supply of health services if they are not used [56], so their success will depend on promoting access to quality services and synergies between effective supply and demand interventions [57–59]. These strategies include the improvement in population and/or geographical coverage of available services, the allocation of subsidies to cover indirect costs (i.e., transportation costs) involved in seeking health services [60], especially among the poorest and geographically dispersed populations, stimulate the use of services by delivering cash [61] and non-monetary transfers [62, 63] aimed at women, the deployment of community strategies [64–67] on the importance of maternal health (e.g. through educational campaigns) and improving the quality of care with a rights perspective and without discrimination [68]. These strategies should rescue the importance of key community actors, social support networks, and their role as promoters of sexual and reproductive education, timely and safe access to contraceptive methods, emergency oral contraception, and safe abortion, as well as support for the identification of obstetric complications and contact and frequent use of health services [69]. Implementing these strategies requires an endowment and composition of human resources trained with high levels of quality, committed to their role as agents of improvement of the populations’ health, particularly of the humanized-holistic care of childbirth, including the prevention of unsafe abortion. It is essential to train HRH capable of exercising leadership in clinical, community, and managerial action and consider health service users as the center of the health system and rights holders [70, 71].

Achieving effective universal access to maternal health and mitigating the risks of setbacks in the achievements observed in the reduction of the MMR due to the COVID-19 pandemic requires the adjustment of social policy innovations, including the rules of allocation and execution of resources for health. Health policies should be designed with a comprehensive perspective to effectively mitigate the structural segmentation of most LAC health systems. In the same way, it is essential to counteract those mechanisms that allow the reproduction of structures of social inequality. The health system must promote human rights, stewardship rights, and effective access to quality health services based on civil rights criteria. This involves the implementation of public policies sensitive to gender and health rights—issues that are pending in the implementation of political agendas in the region.

In sum, this study provides evidence that increasing government health expenditures and human resources for health, including skilled birth attendants, are necessary but insufficient conditions to reduce maternal mortality effectively. It underscores the need for evidence-based health policies that address the structural determinants of maternal mortality based on a nuanced understanding of the needs of the most vulnerable populations in Latin America and the Caribbean. Finally, innovative health policies should be designed with a health equity perspective and promote women-centered approaches that provide respectful care based on human rights, gender, and interculturality.

## Data Availability

Final database underlying this study have been uploaded to figshare and are freely accessible using the following link: 10.6084/m9.figshare.21983516.

## Abbreviations

MMR: Maternal Mortality Ratio
MDGs: Millennium Development Goals
GHE: Government Health Expenditure
GDP: Gross Domestic Product
HRH: Human Resources for Health
SDGs: Sustainable Development Goals
UHC: Universal Health Coverage
LAC: Latin American and Caribbean Region
GBD: Global Burden of Disease
SBA: Skilled Birth Attendance
SDI: Socio-Demographic Index

## Declarations

### Memorial dedication

We dedicate this manuscript to our colleague and friend Sandra Sosa-Rubí, PhD, who inspired us in the analysis of equity during her fruitful lifetime, and who passed away in March 2021.

## References

[1] Murray CJL, Frenk J (2000). A framework for assessing the performance of health systems. Bull World Health Organ.

[2] Castro A, Sáenz R, Avellaneda X, Cáceres C, Galvão L, Mas P (2021). The health equity network of the americas: inclusion, commitment, and action. Pan Am J Public Heal.

[3] Kutzin J (2013). Health financing for universal coverage and health system performance: concepts and implications for policy. Bull World Health Organ.

[4] World Health Organization (WHO). Universal health coverage (UHC) [Internet]. 2022 [cited 2022 May 23]. p. 1–4. Available from: https://www.who.int/news-room/fact-sheets/detail/universal-health-coverage-(uhc).

[5] World Health Organization (WHO). Handbook on health inequality monitoring: With a special focus on low-and middle-income countries. 1st ed. Geneva 27, Switzerland: World Health Organization; 2013.

[6] Bhatia A. Monitoring health inequities in low-and-middle-income countries: Who is– and is not– counted and included in government health statistics? [Internet]. Harvard University; 2019. Available from: https://dash.harvard.edu/handle/1/40977038.

[7] Figueras J, Musgrove P, Carrin G, Durán A (2002). Retos para Los Sistemas Sanitarios De Latinoamérica: ¿Qué puede aprenderse de la experiencia europea?. Gac Sanit.

[8] Vieira Machado C, Dias de Lima L (2017). Health policies and systems in Latin America: Regional identity and national singularities. Cad Saúde Pública.

[9] Comisión Económica para América Latina y el Caribe (CEPAL) (2005). Las reformas de salud en América Latina Y El Caribe: Su Impacto en Los principios de la seguridad social.

[10] Campbell OMR, Calvert C, Testa A, Strehlow M, Benova L, Keyes E (2016). The scale, scope, coverage, and capability of childbirth care. Lancet.

[11] Latin America and Caribbean Task Force for Maternal Mortality Reduction (GTR). Overview of the situation of maternal morbidity and mortality: Latin America and the Caribbean [Internet]. Panama, Republic of Panama; 2017. Available from: https://www.everywomaneverychild-lac.org/e/publication/maternal-morbidity-mortality-latin-america-caribbean/.

[12] Crear-Perry J, Correa-de-Araujo R, Lewis Johnson T, McLemore MR, Neilson E, Wallace M (2020). Social and structural determinants of health inequities in maternal health. J Women’s Heal.

[13] Organisation for Economic Co-operation and Development (OECD)/The World Bank (WB). Maternal mortality. Heal a glance lat am Caribb 2020. 1st ed. OECD/The World Bank; 2020. pp. 74–5.

[14] Kassebaum NJ, Barber RM, Bhutta ZA, Dandona L, Gething PW, Hay SI (2016). Global, regional, and national levels of maternal mortality, 1990–2015: a systematic analysis for the global burden of Disease Study 2015. Lancet.

[15] Hamal M, Dieleman M, De Brouwere V, de Cock Buning T (2020). Social determinants of maternal health: a scoping review of factors influencing maternal mortality and maternal health service use in India. Public Health Rev.

[16] Regional Task Force for Maternal Mortality Reduction (GTR). Interagency strategic consensus for the reduction of maternal morbidity and mortality: Strategic guidance for the 2020–2030 decade. Panama, Republic of Panama; 2021.

[17] Jones GL, Mitchell CA, Hirst JE, Anumba DOC (2022). The Royal College of Obstetricians and gynaecologists. Understanding the relationship between social determinants of health and maternal mortality. BJOG An Int J Obstet Gynaecol.

[18] Campbell OMR, Graham WJ (2006). Strategies for reducing maternal mortality: getting on with what works. Lancet.

[19] World Health Organization (WHO). Trends in maternal mortality 2000 to 2017: Estimates by WHO, UNICEF, UNFPA, World Bank Group and the United Nations Population Division [Internet]., Geneva PP - Geneva: World Health Organization; 2019. Available from: https://apps.who.int/iris/handle/10665/327596.

[20] World Health Organization (WHO). Trends in maternal mortality 1990 to 2015: Estimates by WHO, UNICEF, UNFPA, World Bank Group and the United Nations Population Division., Ginebra 27, Suiza; 2015. Report No.: WHO /RHR/15.23.

[21] Castro A (2020). Maternal and child mortality worsens in Latin America and the Caribbean. Lancet.

[22] Serván-Mori E, Juárez-Ramírez C, Meneses-Navarro S, Heredia-Pi I, Armenta-Paulino N, Orozco-Núñez E et al. Ethnic disparities in effective coverage of maternal healthcare in Mexico, 2006–2018: a decomposition analysis. Sex Res Soc Policy. 2022.

[23] Diana G, Guillermina M, Ñopo H. El Coronavirus y los retos para el trabajo de las mujeres en América Latina [Internet]. New York; 2020. Report No.: No. 18. Available from: https://www.latinamerica.undp.org/content/rblac/en/home/library/crisis_prevention_and_recovery/el-coronavirus-y-los-retos-para-el-trabajo-de-las-mujeres-en-ame.html.

[24] McGranahan M, Nakyeyune J, Baguma C, Musisi NN, Nsibirwa D, Sekalala S (2021). Rights based approaches to sexual and reproductive health in low and middle-income countries: a systematic review. PLoS ONE.

[25] Mujica OJ, Sanhueza A, Carvajal-Velez L, Vidaletti LP, Costa JC, Barros AJD et al. Recent trends in maternal and child health inequalities in Latin America and the Caribbean: analysis of repeated national surveys. Int J Equity Health [Internet]. 2023;22:125. 10.1186/s12939-023-01932-4. 10.1186/s12939-023-01932-4 PMC10314462 37393277

[26] Vargas-Riaño E, Becerril-Montekio V, Becerra-Posada F, Tristán M. Maternal health research outputs and gaps in Latin America: reflections from the mapping study. Global Health [Internet]. 2017;13:74. 10.1186/s12992-017-0300-2. 10.1186/s12992-017-0300-2 PMC5604511 28923096

[27] Restrepo-Méndez MC, Barros AJD, Requejo J, Durán P, Serpa LA, de F, França GVA. Progress in reducing inequalities in reproductive, maternal, newborn, and child health in Latin America and the Caribbean: an unfinished agenda. Restrepo-Méndez MC, editor. Rev Panam Salud Publica;38(1),jul 2015 [Internet]. 2015; Available from: https://iris.paho.org/bitstream/handle/10665.2/10003/v38n1a3.pdf?sequence=1. 26506316

[28] Murray CJL, Lopez AD (2017). Measuring global health: motivation and evolution of the global burden of Disease Study. Lancet.

[29] Stevens GA, Alkema L, Black RE, Boerma JT, Collins GS, Ezzati M (2016). Guidelines for accurate and transparent health estimates reporting: the GATHER statement. Lancet.

[30] Murray CJL, Lopez AD (2013). Measuring the global burden of Disease. N Engl J Med.

[31] Global Burden of Disease (GBD) Collaborative Network. Global Burden of Disease Study 2019 (GBD 2019) socio-demographic index (SDI) 1950–2019 [Internet]. 2020 [cited 2021 Dec 27]. Available from: http://ghdx.healthdata.org/record/ihme-data/gbd-2019-socio-demographic-index-sdi-1950-2019.

[32] Dalenius T, Hodges J (1959). Minimum variance stratification. J Am Stat Assoc.

[33] Cox NJ (2005). Speaking stata: smoothing in various directions. Stata J.

[34] Royston P (2017). Model selection for univariable fractional polynomials. Stata J.

[35] Villanueva-Egan LA, Schiavon-Ermani R (2013). Intervenciones latinoamericanas basadas en evidencia para reducir la mortalidad materna. Rev CONAMED.

[36] Rodríguez-Aguilar R (2018). Maternal mortality in Mexico, beyond millennial development objectives: an age-period-cohort model. PLoS ONE.

[37] Del Carpio Ancaya L (2013). Situación De La mortalidad materna en El Perú, 2000–2012. Rev Peru Med Exp Salud Publica.

[38] Avila-Jaquez C (2019). Disminución De La mortalidad materna en Perú y El enfoque de capacidades. Convergencia.

[39] Nureña CR (2009). Incorporación Del enfoque intercultural en El sistema de salud peruano: La atención Del parto vertical. Rev Panam Salud Publica.

[40] Rubio M, Díaz JJ, Jaramillo M (2009). El Impacto De PARSalud Sobre La Calidad De La atención De Salud materna entre la población indígena.

[41] MacDonald T, Dorcely O, Ewusie JE, Darling EK, Moll S, Mbuagbaw L. The effect of a new maternity unit on maternal outcomes in rural Haiti: An interrupted time series study. BMC Pregnancy Childbirth [Internet]. 2021;21:601. 10.1186/s12884-021-04062-3. 10.1186/s12884-021-04062-3 PMC8418005 34481461

[42] Every mother counts. Impacts on maternal ealth in Haiti [Internet]. Haiti A Deep. drive. 2022 [cited 2022 Apr 7]. Available from: https://everymothercounts.org/grants/haiti-a-deeper-dive/#:~:text=Maternal mortality in Haiti has, childbirth and pregnancy related causes.

[43] Comisión Económica para América Latina y el Caribe (CEPAL) (2021). Balance preliminar de las economías de América Latina Y El Caribe 2020.

[44] Berger MC, Messer J. Public financing of health expenditures, insurance, and health outcomes. Appl Econ [Internet]. 2002;34:2105–13. 10.1080/00036840210135665.

[45] Burgos Bizama A. Políticas públicas en América Latina para la reducción de la mortalidad materna, 2009–2014 [Internet]. Santiago de Chile; 2015. Report No.: 112. Available from: https://www.cepal.org/es/publicaciones/39303-politicas-publicas-america-latina-la-reduccion-la-mortalidad-materna-2009-2014.

[46] Ooms G, Stuckler D, Basu S, McKee M (2010). Financing the Millennium Development Goals for health and beyond: sustaining the big push. Global Health.

[47] World Health Organization (WHO). WHO recommendations: Intrapartum care for a positive childbirth experience. 1st ed. Geneva 27, Switzerland: World Health Organization; 2018. 30070803

[48] Sassi F, Le Grand J, Archard L (2001). Equity versus efficiency: a dilemma for the NHS. BMJ.

[49] Reidpath DD, Olafsdottir AE, Pokhrel S, Allotey P (2012). The fallacy of the equity-efficiency trade off: rethinking the efficient health system. BMC Public Health.

[50] Hutson M, Sacoby W (2011). The role of community-based strategies in addressing metropolitan segregation and racial health disparities. Community Dev.

[51] Rushton G (1988). The Roepke lecture in economic geography location theory, location-allocation models, and service development planning in the third world. Econ Geogr.

[52] Carr-Hill R, Hardman G, Martin S, Peacock S, Sheldon TA, Smith PC. A new formula for distributing hospital funds in England. Interfaces (Providence) [Internet]. 1997;27:53–70. Available from: http://www.jstor.org/stable/25062211.

[53] Quesnel-Barbet A, Nuttens MC, Aublet-Cuvellier B, Warembourg H, Prat A, Thumerelle PJ et al. Modelling a regional reorganization of cardiovascular surgery provision. Health Place [Internet]. 2005;11:283–92. Available from: http://www.sciencedirect.com/science/article/pii/S1353829204001157. 10.1016/j.healthplace.2004.06.007 15774334

[54] Barrios EB (2008). Infrastructure and rural development: Household perceptions on rural development. Prog Plann.

[55] Frenk J (2015). Leading the way towards universal health coverage: a call to action. Lancet.

[56] Figueroa ME, Kincaid DL. Social, cultural and behavioral correlates of household water treatment and storage [Internet]. Cent. Publ. HCI 2010-1 Heal. Commun. Insights. Baltimore, MD 21202; 2010. Available from: http://ccp.jhu.edu/wp-content/uploads/Household-Water-Treatment-and-Storage-2010.pdf.

[57] Gaarder MM, Glassman A, Todd JE (2010). Conditional cash transfers and health: unpacking the causal chain. J Dev Eff.

[58] Ranganathan M, Lagarde M (2012). Promoting healthy behaviours and improving health outcomes in low and middle income countries: a review of the impact of conditional cash transfer programmes. Prev Med (Baltim).

[59] Serván-Mori E, Cerecero-García D, Heredia-Pi I, Pineda-Antúnez C, Sosa-Rubí S, Nigenda G (2019). Improving the effective maternal-child health care coverage through synergies between supply and demand-side interventions: evidence from Mexico. J Glob Health.

[60] Alfonso YN, Bishai D, Bua J, Mutebi A, Mayora C, Ekirapa-Kiracho E (2015). Cost-effectiveness analysis of a voucher scheme combined with obstetrical quality improvements: quasi experimental results from Uganda. Health Policy Plan.

[61] Feldman BS, Zaslavsky AM, Ezzati M, Peterson KE, Mitchell M (2009). Contraceptive use, birth spacing, and autonomy: an analysis of the Oportunidades program in rural Mexico. Stud Fam Plann.

[62] Banerjee AV, Duflo E, Glennerster R, Kothari D (2010). Improving immunisation coverage in rural India: clustered randomised controlled evaluation of immunisation campaigns with and without incentives. Brithis Med J.

[63] Wang P, Connor AL, Guo E, Nambao M, Chanda-Kapata P, Lambo N (2016). Measuring the impact of non‐monetary incentives on facility delivery in rural Zambia: a clustered randomised controlled trial. Trop Med Int Heal.

[64] Haroon S, Das JK, Salam RA, Imdad A, Bhutta ZA (2013). Breastfeeding promotion interventions and breastfeeding practices: a systematic review. BMC Public Health.

[65] Prost A, Colbourn T, Seward N, Azad K, Coomarasamy A, Copas A (2013). Women’s groups practising participatory learning and action to improve maternal and newborn health in low-resource settings: a systematic review and meta-analysis. Lancet.

[66] Adam MB, Dillmann M, Chen M, Mbugua S, Ndung’u J, Mumbi P (2014). Improving maternal and newborn health: effectiveness of a community health worker program in rural Kenya. PLoS ONE.

[67] Naugle DA, Hornik RC (2014). Systematic review of the effectiveness of mass media interventions for child survival in low-and middle-income countries. J Health Commun.

[68] Castro A, Savage V, Kaufman H (2015). Assessing equitable care for indigenous and afrodescendant women in Latin America. Pan Am J Public Heal.

[69] Rifkin SB (2014). Examining the links between community participation and health outcomes: a review of the literature. Health Policy Plan.

[70] Campbell J, Buchan J, Cometto G, David B, Dussault G, Fogstad H (2013). Human resources for health and universal health coverage: fostering equity and effective coverage. Bull World Health Organ.

[71] Dussault G, Dubois CA (2003). Human resources for health policies: a critical component in health policies. Hum Resour Health.

[72] Global Burden of Disease Collaborative (GBD). Network. Global burden of disease study 2019 (GBD 2019) results [Internet]. 2021 [cited 2021 Nov 5]. Available from: http://ghdx.healthdata.org/gbd-results-tool.

